# Radiometric and spectral characterization of multipanel planar light-emitting devices: a generalizable irradiance mapping approach for dose estimation

**DOI:** 10.1117/1.JBO.31.5.055001

**Published:** 2026-05-19

**Authors:** Emmanuel Gerelli, Georges Wagnières, Jaroslava Joniová

**Affiliations:** Institute of Physics, Swiss Federal Institute of Technology (EPFL), Laboratory for Functional and Metabolic Imaging, Lausanne, Switzerland

**Keywords:** light dosimetry, photobiomodulation, ATP38®, radiometric characterization, cell migration, PpIX

## Abstract

**Significance:**

Accurate spectral and radiometric characterization of photobiomodulation (PBM) devices is essential for reliable dosimetry and reproducible therapeutic outcomes. Complex, multilight-emitting diode (LED) systems produce heterogeneous irradiance patterns that complicate dose estimation, underscoring the need for standardized quantitative methods.

**Aim:**

To develop and validate a generalizable methodology for the spectral and radiometric characterization of multipanel and multi-LED PBM devices, demonstrated using the ATP38® system.

**Approach:**

Spectral properties were measured using an optical fiber–based spectrometer. The radiometric properties were determined by measuring 2D irradiance maps acquired at a 4-cm working distance from a single panel at six wavelengths. A critical element of the method was correcting raw irradiance data for the detector’s nonideal angular response, modeled by a $\cos^{m}(\theta )$ function, with m determined for each wavelength. The corrected maps were fitted using double 2D Gaussian surfaces, enabling the generation of continuous irradiance distributions and their extrapolation to other working distances through homothety. This framework also allowed simulation of irradiance maps for multipanel configurations. To validate the physical characterization, *in vitro* experiments on normal human epidermal keratinocytes compared the biological effects of three illumination protocols applied at two panel locations. PBM effects were assessed by measuring the endogenously produced protoporphyrin IX fluorescence intensity and by the scratch test assay.

**Results:**

Six emission peaks were identified at 454, 525, 594, 627, 729, and 842 nm. The detector angular correction improved the accuracy of the radiometric characterization, and Gaussian modeling of the irradiance enabled its prediction at different working distances. Biological responses correlated with spatial irradiance variations.

**Conclusions:**

This framework enables quantitative characterization of the ATP38® device spectral and radiometric properties, supporting accurate light propagation modeling and dosimetry optimization.

## 1 Introduction

Precise estimation of the therapeutic irradiance and light dose delivered by light-emitting devices is paramount for ensuring the efficacy and safety of phototherapy applications. This requires, as a foundational step, a comprehensive characterization of the device’s radiometric and spectral properties. Without a precise understanding of the light emission, including its spatial distribution and spectral composition, the ability to predict and control the dose received by biological tissues is severely compromised.^1^^,^^2^ This challenge is particularly pronounced for devices featuring complex illumination geometries, such as those with multiple LEDs emitting diverging light beams.

Photobiomodulation (PBM) devices based on arrays of light-emitting diodes (LEDs) are increasingly used in dermatological and regenerative applications. However, their biological effects depend strongly on the spatial distribution of irradiance delivered to the tissue, which is often insufficiently characterized. A rigorous physical description of these illumination patterns is therefore necessary to better understand and interpret associated biological responses.

To address this need, the present study presents a methodology for the radiometric and spectral characterization of a multipanel LED-based PBM device (ATP38®). It should be noted that the primary goal of this work is to characterize the device’s spectral and radiometric properties, not to develop or optimize such a PBM system. By combining spatial irradiance mapping with mathematical modeling, this approach enables the prediction of light distribution under different clinically relevant configurations and working distances. The wavelengths implemented in the ATP38® device were selected by the manufacturer based on prior work, particularly studies by Karu and colleagues, which identified spectral regions associated with mitochondrial photoacceptors and cellular metabolic modulation in PBM processes.^3^

Mitochondria are widely recognized as the primary sites of interaction for PBM,^4^^–^^7^ prompting us to explore its influence on the endogenous production of protoporphyrin IX (PpIX). PpIX is a fluorescent intermediate in the mitochondrial heme biosynthesis pathway and is closely tied to mitochondrial metabolic processes. Because it is produced directly within the mitochondria, PpIX can serve as a noninvasive and direct indicator of mitochondrial metabolic activity.^4^ Furthermore, heme, of which PpIX is a key precursor, is essential for numerous biological functions, including oxygen transport, energy production, and enzymatic catalysis.^8^ To stimulate PpIX accumulation, we used its natural precursor, five-aminolevulinic acid (ALA), a clinically approved and widely used prodrug that naturally enters the heme synthesis pathway. When administered exogenously, ALA leads to a robust, time-dependent increase in intracellular PpIX levels, typically sustained for several hours.^4^

To connect physical characterizations of the ATP38@ with biological effects, we investigated the response of normal human epidermal keratinocytes (NHEK) exposed to three manufacturer-defined PBM protocols. Using this model, we examined how spatial variations in irradiance influence cellular metabolism and migration; PpIX fluorescence was measured following PBM, and a scratch assay was performed to evaluate effects on keratinocyte migration, providing a direct link between the physical properties of complex PBM illumination systems and measurable biological outcomes.^9^

## 2 Material and Methods

### 2.1 Device Description

**Fig. 1:**
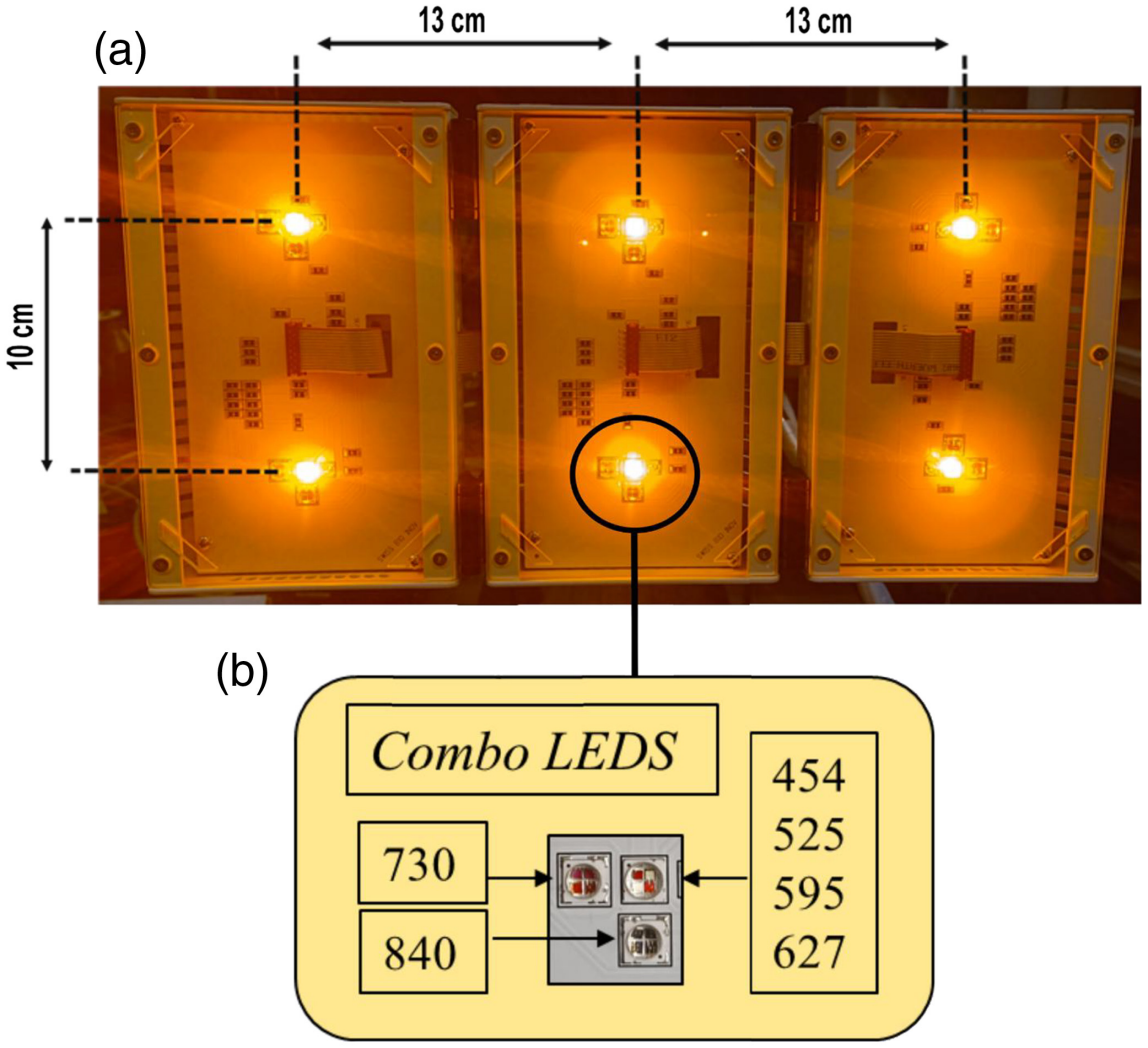
Overview of the ATP38® device illumination panels and internal source configuration. (a) Images of the three illumination panels of the ATP38® device, showcasing their physical appearance. (b) Detailed schematic of one “center source,” which is the fundamental light-emitting unit within each panel.

The ATP38® device (Swiss Bio Inov, Moudon, Switzerland) consists of three identical panels designed for phototherapy applications [Fig. 1(a)]. Each panel incorporates two “center sources,” with each source composed of three individual LEDs or groups of LEDs [Fig. 1(b)]. The device emits light at six distinct wavelengths labeled: 454 nm (blue), 525 nm (green), 595 nm (yellow), 627 nm (red), 730 nm, and 840 nm. These wavelengths are generated by three juxtaposed LED groups within each “center source.” Specifically, one LED group emits the blue, green, yellow, and red light. A separate LED, located ∼1  cm to the left of this primary group in Fig. 1(b), emits at 730 nm, whereas another LED, positioned ∼1  cm below the primary group, emits at 840 nm. These LEDs are protected by 12  cm×18.5  cm transparent polycarbonate plates visible in Fig. 1(a).

Because the three panels are mechanically identical and separated by fixed interpanel distances, the optical characterization was performed on a single panel and subsequently extrapolated to the multipanel configuration. This approach avoids interpanel optical crosstalk during measurements while allowing reconstruction of the full device emission by spatial translation and superposition of the single-panel data.

### 2.2 Spectral Characterization

The spectral properties of the ATP38® illumination system were characterized using a spectrometer (USB2000, Ocean Optics, Orlando, FL, USA) coupled to a quartz optical fiber (Thorlabs, Newton, NJ, USA; 400-μm-core diameter; numerical aperture 0.22). The distal tip of the optical fiber was positioned perpendicularly at the center of a single illumination panel, at a fixed distance of 30 cm, to collect the emitted light.

Spectral measurements were performed in continuous acquisition mode, with each wavelength assessed sequentially. For each acquisition, the signal represented the average of 20 individual spectra. The integration time was set to 3 ms, with a target maximum signal intensity of ∼4000 counts. The background signal was subtracted from all measurements. This background signal was obtained in the dark with the same acquisition parameters.

Following background correction, raw spectra were normalized to their respective maximum intensity. The full width at half maximum (FWHM) was determined for each normalized spectrum.

This spectral characterization was conducted on one of the central light sources of the ATP38® device, which integrates six distinct LED groups arranged side-by-side. These groups emit in the blue (454 nm), green (525 nm), yellow (594 nm), red (627 nm), near-infrared (729 nm), and infrared (842 nm) regions. This detailed spectral profiling serves as the foundation for accurate dose estimation and light propagation modeling in biological tissues.^10^

### 2.3 Irradiance Measurement Setup

The irradiance, *E* [$\mathrm{mW}/{\mathrm{cm}}^{2}$], produced by a single panel of the ATP38® device was measured using a dedicated experimental setup. A single panel was securely mounted on an optical table, ensuring its surface was precisely perpendicular to the table. A calibrated planar power meter (Thorlabs, Newton, NJ, USA; Model PM160–400/1000 nm spectral range–20 nW/200 mW power range–$0.71\;{\mathrm{cm}}^{2}$ sensitive surface area) was positioned on a 2D translation stage (Newport, Irvine, CA, USA) at a fixed distance of 4 cm from the external surface of the panel’s transparent polycarbonate plate, which is the output window of the ATP®38. The translation stage facilitated precise movement in a plane parallel to this plate across a range of 26 cm along the *X*-axis and 28 cm along the *Y*-axis. To isolate the light contribution from the single active panel, the other two ATP38® panels were completely obscured with a metallic black sheet. This configuration ensured that the recorded irradiance maps corresponded exclusively to one panel, which could then be used to model the complete three-panel arrangement. The reference position (X=Y=0  cm) was established by aligning the center of the power meter’s detector head precisely with the midpoint between the two combo LEDs of the illuminated panel. Irradiance measurements were systematically collected at 1 (“close” to the LEDs group) or 2 cm (“far” from the LEDs group) intervals along both the X and Y axes, whereas the ATP38® was emitting light continuously. The accuracy of the X/Y displacements was verified to be submillimetric. The control of the working distance, *Z*, was identified as the primary source of experimental error.

### 2.4 Angular Response Correction of the flat power meter

Given that the Thorlabs detector exhibits a nonideal angular response (i.e., not strictly proportional to cos θ), a correction procedure was implemented. The detector’s angular response was characterized by mounting it on a goniometer (Newport, Irvine, CA, USA) and illuminating it with a broad, collimated light beam. This light beam was much larger than the photosensitive surface of the detector. The relative power measured by the detector was recorded every 5 deg at angles ranging from 0 deg to 90 deg. This characterization was performed for each wavelength emitted by the ATP38® device. The angular response was modeled by a function proportional to $\cos^{m}(\theta )$, where m is a fitting coefficient determined through a curve-fitting procedure. Raw irradiance values were subsequently divided by an angular correction map, calculated for the two distinct light sources ($E_{1}$ and $E_{2}$) of the panel (assuming identical angular properties), to derive the corrected irradiance maps. More precisely, we computed the angles $\theta_{x1y1}$ and $\theta_{x2y2}$ for each X−Y position of the power meter for the two light sources $E_{1}$ and $E_{2}$. The resulting correction map was therefore given by $\cos^{m}\theta_{x1\;y1}+\cos^{m}\theta_{x2\;y2}$.

### 2.5 Irradiance Map Fitting and Simulation

The corrected 2D irradiance maps of the single panel were subjected to a continuous function fitting using a double 2D Gaussian surface model. This fitting was executed using the Levenberg-Marquardt algorithm, as implemented in OriginPro 2018 analysis software (OriginLab Corporation, Northampton, MA, USA). The fitted area for a single panel encompassed X=Y=−20  cm; 20 cm, with a target spatial resolution of 0.1 mm for the generated maps. A key application of these fitted maps is their utility in generating irradiance distributions at any arbitrary working distance from the source through a simple homothecy (scaling) transformation. This scaling approach enables the prediction of irradiance distributions at clinically relevant working distances without requiring additional physical measurements. Furthermore, the irradiance map generated by the three panels of the ATP38® operating together in a “planar” configuration was simulated. This was achieved by summing the contributions of three identical single-panel maps, translated by 0, 13, and 26 cm, respectively (Fig. 1), under the assumption that all three panels possess identical optical properties.

### 2.6 Cell Culture

Primary normal human epidermal keratinocytes, isolated from the epidermis of juvenile foreskin or adult skin from pooled donors, were purchased from PromoCell (Heidelberg, Germany; Cat. No. C-12006). Cells were maintained in Keratinocyte Growth Medium 2 (KGM2; PromoCell), a serum-free cell culture medium for keratinocytes from the epidermis supplemented with 4 mL/L bovine pituitary extract, 0.125 ng/mL epidermal growth factor (recombinant human), 5  μg/mL insulin (recombinant human), 0.33  μg/mL hydrocortisone, 0.39  μg/mL epinephrine, 10  μg/mL transferrin (recombinant human), 0.06 mM ${\mathrm{CaCl}}_{2}$, 50 I.U./mL penicillin, and 50  μg/mL streptomycin. Cells were incubated at 37°C in the dark, in the presence of 5% ${\mathrm{CO}}_{2}$ (Sanyo ${\mathrm{CO}}_{2}$ incubator, Osaka, Japan) until the confluence reached 90%.

Before the experiments, cells were washed and detached from the flasks with DetachKit (PromoCell) before centrifugation. After 5 min of centrifugation at 1200 relative centrifugal force (Renggli Laboratory systems, Rotkreuz, Switzerland), NHEK cells were seeded in the single Falcon® Petri Dishes (Corning, NY, USA) or 12-well plates (Corning, NY, USA) and incubated for 24 h at 37°C in 5% ${\mathrm{CO}}_{2}$. Because primary keratinocytes exhibit donor-dependent variability in attachment and proliferation, experiments were standardized based on the degree of confluency at the time of treatment rather than a fixed initial seeding density. For experiments performed in Petri dishes, cells were used at ∼50% confluency at the time of PBM exposure. For scratch assays in 12-well plates, cells were cultured to full confluency to establish a continuous monolayer before wounding.

### 2.7 PBM Irradiation Protocol

Three PBM conditions were applied using the ATP38® light source (Swiss Bio Inov, Moudon, Switzerland). These conditions were selected in agreement with Swiss Bio Inov (Table 1).

**Table 1:** Detailed PBM illumination protocols defined in the ATP38® for the respective studied PBM conditions. Duty cycles were 99% for the first two protocols.

Protocol name	Wavelength (nm)	454	525	594	627	729	842
Anti-inflammatory	Illumination time (s)	151	182	241	244	162	219
	Light dose ($J/{\mathrm{cm}}^{2}$)	2	1	0.8	2	4	4
	Frequency (Hz)	5	5	5	5	5	5
Analgesic	Illumination time (s)	151	182	241	244	162	219
	Light dose ($J/{\mathrm{cm}}^{2}$)	2	1	0.8	2	4	4
	Frequency (Hz)	70	70	70	70	70	70
Wound healing	Illumination time (s)	224	180	239	242	160	217
	Light dose ($J/{\mathrm{cm}}^{2}$)	3	1	0.8	2	4	4
	Frequency (Hz)	CW	CW	CW	CW	CW	CW

CW, continuous wave.

Petri Dishes (PDs) were inserted into a dedicated homemade holder in such a way that the distance between the bottom surface of the PD and the upper surface of the transparent plate of the ATP38® panels was 4 cm. PBM irradiation was delivered from the bottom. Two illumination positions were investigated: (i) directly above the LED groups and (ii) at the geometrical center of the panel, allowing evaluation of the biological impact of spatially heterogeneous irradiance. After the irradiations, PDs were returned to the incubator for 24 h. Sham controls (no PBM samples) were kept in the dark at all times. Then, ALA was applied for 3 h to induce the production of PpIX.

### 2.8 ALA Administration

Twenty-four hours after PBM, 5-aminolevulinic acid (ALA; Sigma-Aldrich, St. Louis, MI, USA), a precursor of protoporphyrin IX (PpIX) in the heme biosynthesis pathway, was dissolved in Keratinocyte Growth Medium (KGM) and added to NHEK cells at a final concentration of 1 mM. Given that the pH of KGM ranges from 7.2 to 7.4, it remained within the desired range after ALA addition, and no further pH adjustment was required. Cells were incubated with ALA for 3 h under standard culture conditions. Following incubation, cells were washed twice with phosphate-buffered saline (Gibco PBS; Thermo Fisher Scientific, Waltham, MA, USA), and ALA-free KGM was added for subsequent PpIX fluorescence intensity measurements using the microscope described below.

### 2.9 PpIX Fluorescence Imaging and Analysis in NEHK Cells

The endogenous production of PpIX in NEHK cells was recorded with an epifluorescence microscope (Nikon, Labophot-2; Tokyo, Japan) accessorized with a mercury vapor lamp (HbO, 100 W), an air-cooled, slow scan 16-bit CCD camera, and a flat 10X objective (NA: 0.1; Nikon,) with a BV-2A filter cube (excitation: 400 to 440 nm; emission: 470 nm high pass filter; 455 nm dichroic mirror) combined with an additional 610-nm long-pass filter (E610LP filter; Chroma Technology Corp, Bellows Falls, USA) transmitting light above 610 nm.^4^^,^^11^

For the PpIX fluorescence analysis, a semi-automatic pipeline algorithm developed in our lab was used. This pipeline was based on Bradley’s adaptive thresholding method for cell segmentation and quantification of fluorescence intensity in fluorescent microscope images.^12^ This process was implemented using MATLAB [version R2020a (9.8)] and its GUI development environment.^9^ For each condition, fluorescence measurements were obtained from a minimum of three independent experiments, with multiple fields of view analyzed per sample.

### 2.10 Scratch Test Assay

NHEK cells were seeded to confluence in a 12-well plate (Corning, NY, USA) one day before a scratch wound was created with a sterile 200  μL pipette tip. Due to the dimensions of the 12-well plate, the culture surface covered nearly the entire illuminated area of the panel, resulting in simultaneous exposure of all wells to a spatially averaged light field. Six wells were shielded from illumination from the bottom and served as nonirradiated (“no PBM”) controls, whereas the remaining six wells were exposed to PBM. Right after the scratch, the medium was changed, cells were irradiated, and phase contrast images were taken at t=0  h. Well plates were then returned to the incubator. Another sets of images were taken 24 h to observe and measure the remaining denuded area. ImageJ plugin (ImageJ 1.52a software macro; NIH, Bethesda, MD, USA) was used to calculate the percentage of the wound closure surface. Data were expressed as the ratio of the PBM and no PBM samples. The scratch assay was performed in at least three independent experiments, each including both PBM-treated and nonirradiated control wells.

### 2.11 Statistical Analysis

All values are reported as the mean ± SEM. Statistical analysis was carried out using OriginPro 2018. Before analysis, data were checked for normal distribution with the Shapiro-Wilk and Kolmogorov-Smirnov tests, and all data sets were confirmed to be normally distributed. To compare groups, a one-way ANOVA was performed. Tukey’s *post hoc* test was used for multiple comparisons, with statistical significance defined as a *P* value less than 0.05.

## 3 Results

### 3.1 Spectral Characteristics

**Fig. 2:**
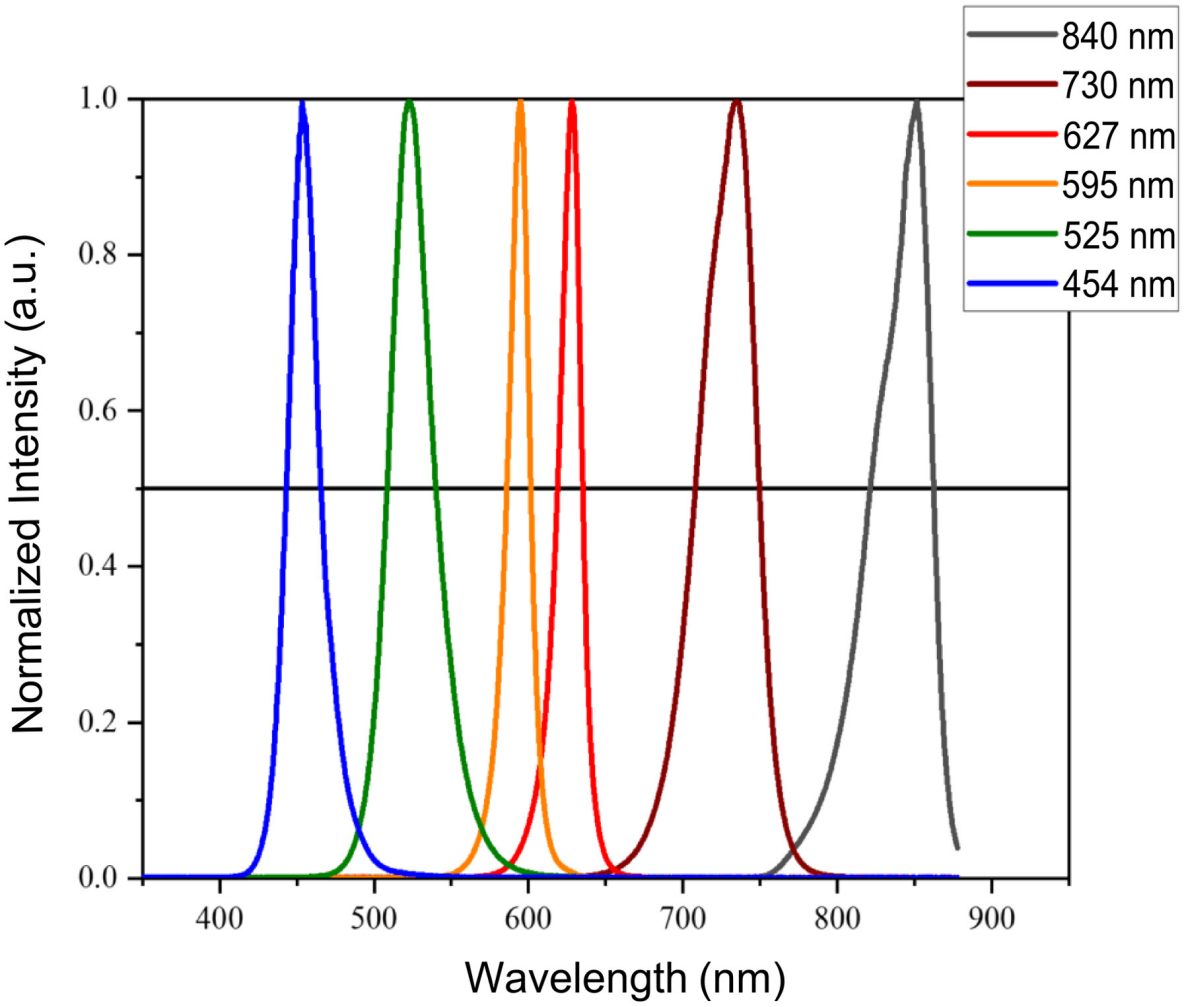
Normalized spectral intensity profiles of the light emitted by the ATP38® device. These graphs display the normalized intensity of light emitted at various wavelengths: blue (454 nm), green (525 nm), orange (595 nm), red (627 nm), brown (730 nm), and gray (840 nm).

The spectral properties of the light emitted by the ATP38® were characterized. Normalized spectra obtained for all emitted wavelengths are presented in Fig. 2. This figure displays the FWHM for each LED emission. The numerical values of these FWHM are detailed in Table 2. The assigned characteristic wavelength values, determined as the mean of the wavelength range at half maximum, were: 454, 525, 594, 627, 729, and 842 nm. For consistency with subsequent documentations, these wavelengths are referred to as 454, 525, 595, 627, 730, and 840 nm, respectively.

**Table 2:** Key wavelength parameters derived from normalized emission spectra.

Peak	$\lambda_{\max}$ (nm)	$\lambda_{\mathrm{FWHM}\_\mathrm{Low}}$ (nm)	$\lambda_{\mathrm{FWHM}\_\mathrm{mid}}$ (nm)	$\lambda_{\mathrm{FWHM}\_\times \max}$ (nm)
1	453.6	442.8	454.0	465.0
2	522.6	508.3	524.5	539.7
3	594.9	585.8	594.3	601.4
4	628.2	618.5	627.4	635.0
5	735.0	707.9	729.0	749.5
6	851.6	821.1	841.7	862.3

This table presents the precise wavelength values extracted from the normalized emission spectra displayed in Fig. 2. For each identified spectral peak, $\lambda_{\max}$ denotes the wavelength corresponding to the maximal intensity. $\lambda_{\mathrm{FWHM}\_\mathrm{Low}}$ and $\lambda_{\mathrm{FWHM}\_\times \max}$ represent the lower and upper wavelengths, respectively, at 50% of the normalized maximal intensity, thereby defining the boundaries of the FWHM. $\lambda_{\mathrm{FWHM}\_\mathrm{mid}}$ is the mean wavelength calculated from $\lambda_{\mathrm{FWHM}\_\mathrm{Low}}$ and $\lambda_{\mathrm{FWHM}\_\times \max}$, serving as the assigned characteristic wavelength for each emission band.

### 3.2 Irradiance Measurements and Angular Response

**Fig. 3:**
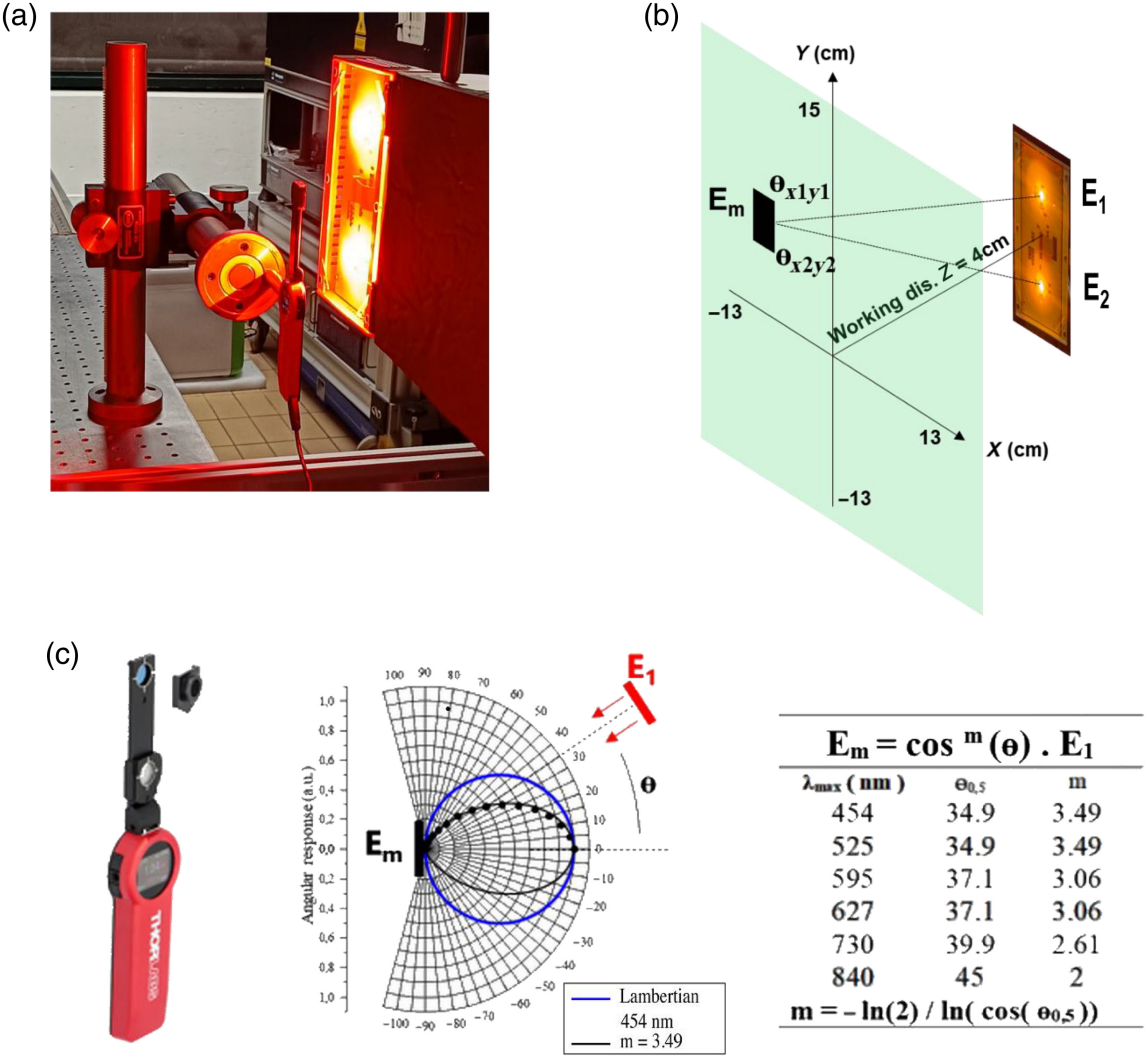
Irradiance measurement setup and values of the detector angular response. (a) Illustration of the experimental setup showing the calibrated Thorlabs power meter on the *XY* translation stage, positioned at 4 cm from the light source panel. (b) Schematic detailing the measurement grid (*X*: {−13 to 13} cm, Y: {−13 to 15} cm) and the angles ($\theta E_{1},\theta E_{2}$) used to correct the angular response of the detector when measuring light emitted by the sources $E_{1}$ and $E_{2}$​. (c) Illustrative angular response of the detector (black points) at 454 nm compared with an ideal Lambertian profile (blue curve). The table provides $\theta_{0.5}$, the angles at which the signal is equal to half of its maximal value, and the fitted parameter “m” for various wavelengths.

The experimental setup developed to measure the irradiance, E [$\mathrm{mW}/{\mathrm{cm}}^{2}$], of a single panel is depicted in Figs. 3(a) and 3(b). During these measurements, typical power values recorded by the Thorlabs detector ranged from a baseline of 0.3  μW to a maximum of 20 mW. These measurements generated 2D raw data matrices for each emitted wavelength.

To account for the nonideal angular response of the Thorlabs detector, its angular sensitivity was characterized. Figure 3(c) presents the measured relative powers for different angles. This angular response of the detector was modeled by a function proportional to $\cos^{m}(\theta )$ to generate the respective correction values for each position of the detector and for each source $E_{1}$ or $E_{2}$. For instance, at 454 nm, the angle at which the signal was half the maximal value ($\theta_{0.5}$) was determined to be 34.9 deg, which corresponded to an “m” value of 3.49 (see Fig. 3). The raw irradiance values were then divided by these correction values. In this procedure, the two distinct light sources ($E_{1}$ and $E_{2}$) in the panel were treated as identical, point-like sources. This is a reasonable assumption because the measurements were taken at source–detector distances larger or equal to 40 mm, whereas their emitting surfaces are on the order of a few tenths of a millimeter. Consequently, the irradiance produced by each source at the distance *d* from the detector was considered as proportional to $1/d^{2}$.

The relative error affecting irradiance measurements, considering a conservative estimate of ±1  mm as error affecting the working distance Z, was ∼±8%, given the $1/Z^{2}$ dependence of irradiance and the “measurement uncertainty” of the detector, according to the specifications given by the manufacturer.

### 3.3 Irradiance Map Fitting and Derived Parameters

**Fig. 4:**
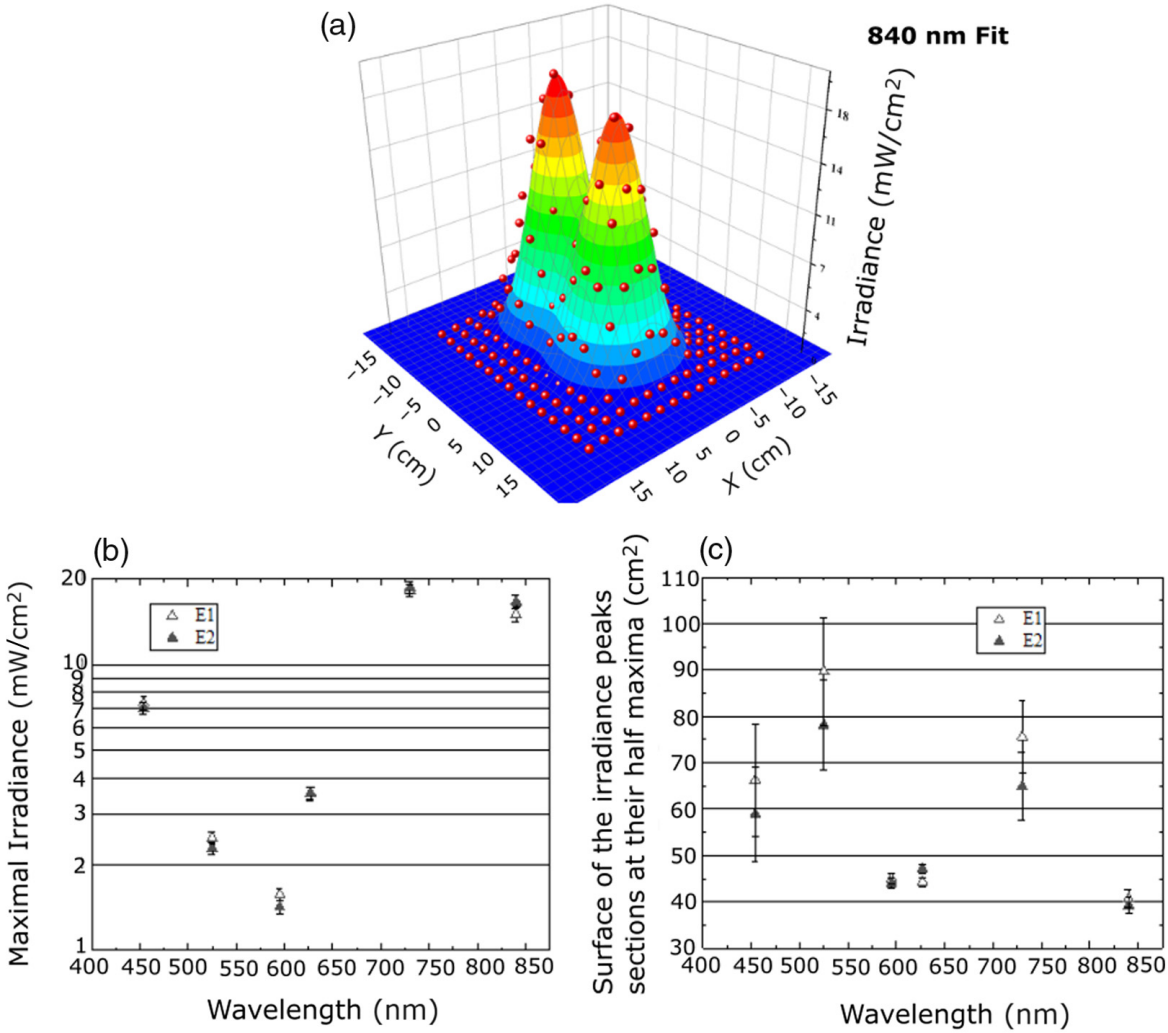
Illustrative fitted irradiance maps and derived radiometric parameters measured at a 4-cm source–detector distance: (a) Superposition of the corrected irradiance data points (red points, 2-cm spatial resolution) with the corresponding 2D double Gaussian surface fit. (b) Maximal irradiance values for the sources $E_{1}$ and $E_{2}$ derived from the fitted surfaces for all measured wavelengths. (c) Surface of the irradiance peaks at their half maximum for $E_{1}$ and $E_{2}$, derived from the fitted surfaces, for all wavelengths.

The corrected 2D irradiance maps were subsequently fitted with a continuous function using a double 2D Gaussian surface model. An illustrative example of this fitted surface is presented in Fig. 4(a) for the measurements performed at 840 nm.

The goodness of fit ($r^{2}$) for all wavelengths consistently exceeded 0.95 for the continuous fitted maps. These fitted maps provide a comprehensive representation of the irradiance distribution. From these fitted maps, key radiometric parameters were extracted. Figure 4(b) illustrates the maximal irradiance values obtained for each wavelength, whereas Fig. 4(c) presents the surfaces of the irradiance peak sections at their half maxima of the irradiance peaks at all wavelengths considered. The total power of the light emitted by each LED can be estimated from Fig. 4, assuming that the irradiance distributions can be fitted with circular Gaussian profiles. If *E* is the maximal irradiance and *S* the surface of the irradiance peaks sections at their half-maximum, the total power is P=(E·S)/ln 2. This leads to powers of 643, 277, 95, 221, 1855, and 827 mW at 454, 525, 595, 627, 730, and 840 nm, respectively.

### 3.4 Irradiance Maps of Single and Multipanel Configurations

**Fig. 5:**
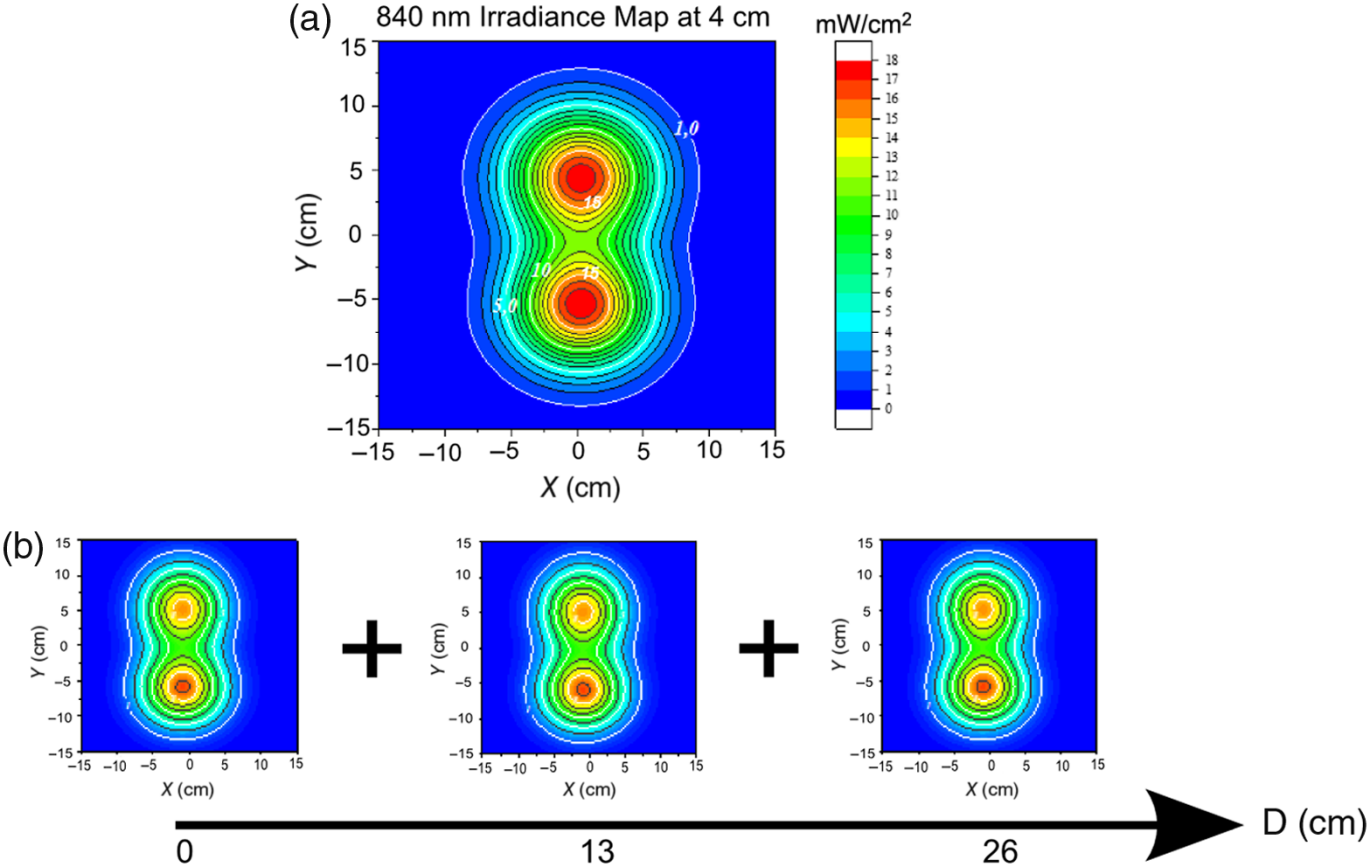
Single-panel irradiance map at 840 nm and multipanel construction. (a) Illustrative corrected irradiance map of a single ATP38® panel measured at a 4-cm working distance. White iso-contour lines indicate irradiance values of 1, 5, 10, and $15\;\mathrm{mW}/{\mathrm{cm}}^{-2}$. (b) Illustration of the method used to construct the irradiance map generated by three illumination panels from the data of a single panel.

Based on the established methodology described above, Fig. 5(a) displays the irradiance maps generated by a single panel of the ATP38® device at a working distance of 4 cm at 840 nm.

**Fig. 6:**
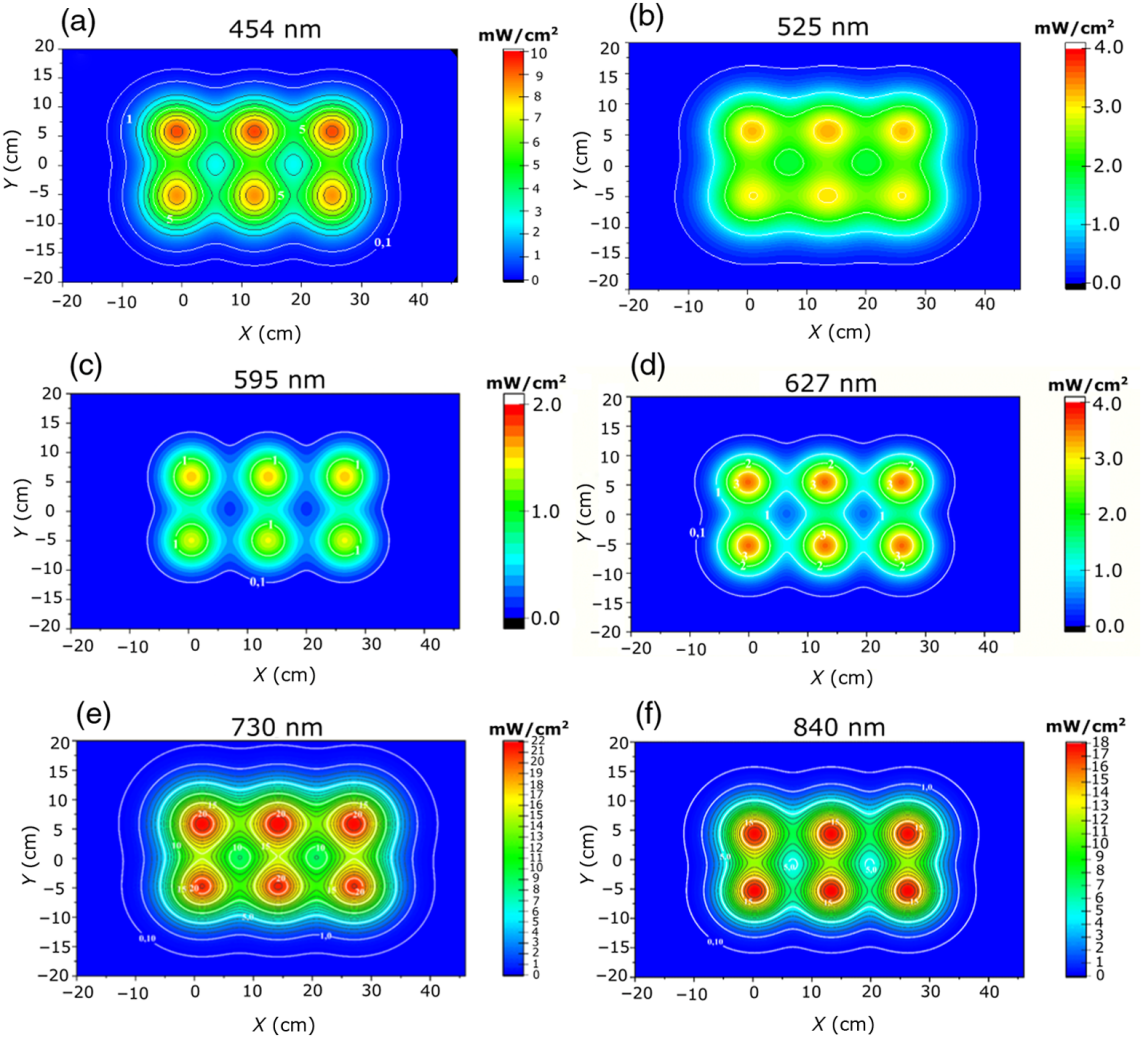
Irradiance maps of the three-panel ATP38® at a 4-cm detector-source distance. Maps are presented for (a) 454 nm, (b) 525 nm, (c) 597 nm, (d) 627 nm, (e) 730 nm, and (f) 840 nm.

To determine the irradiance map for a three-panel ATP38® configuration at this 4 cm distance, the contributions of a single panel were added, translated by 0, 13, and 26 cm [Fig. 5(b)], assuming all three panels possess identical optical properties. In most of these maps, contour lines are presented with a step of $1\;\mathrm{mW}/{\mathrm{cm}}^{2}$. The resulting irradiance map for these three panels at a detector-source distance of 4 cm is presented in Figs. 6(a)–6(f) for each wavelength.

### 3.5 Biological Effects of PBM Protocols

**Fig. 7:**
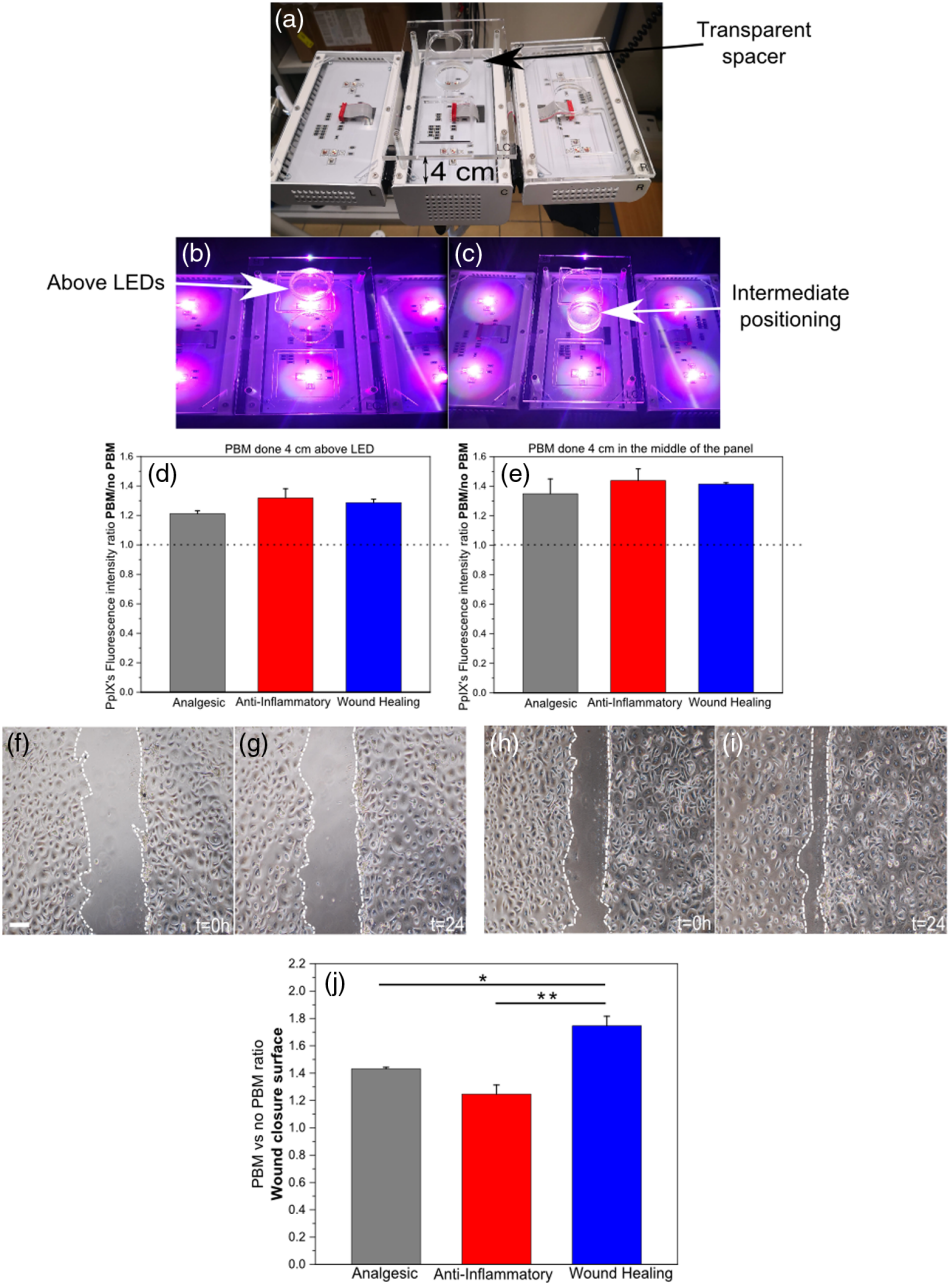
Effects of photobiomodulation on the endogenous production of PpIX and Scratch Test Assays in NHEK. (a)–(c) Illustration of the experimental setup and sample placement. (a) Illustration of the custom 5-mm-thick transparent polycarbonate spacers used to position Petri dishes at a 4-cm working distance from the light source. Holes were managed in these spacers to receive the Petri dishes. (b)–(c) Two distinct sample placements on the ATP38® panel: directly above the LEDs (b) and in the center of the panel (c). (d)–(e) Effects of different PBM protocols on the endogenous PpIX production when samples were placed directly above the LEDs (d) or in the center of the panel (e). The dashed line indicates the value for a sham-treated control. (f)–(j) Representative images of the cell migration (scratch test) assay are shown for no PBM control [(f) and (g) at 0 h or 24 h, respectively] and PBM-treated samples [(h) and (i) at 0 h or 24 h after PBM, respectively], with the scale bar representing 100  μm. (j) Quantification of the effects of the three protocols on cell migration (scratch test), expressed as the ratio of wound-closure percentage for PBM-treated cells to untreated controls after 24 h, corrected for the baseline condition at 0 h. Cells were placed above LEDs. Data are presented as mean ±SEM (n≥3 independent measurements). Statistical significance is indicated as *P<0.05 and **P<0.01.

The biological effects of three distinct PBM protocols were assessed on NHEK cells. The PBM effects on the endogenous PpIX production in cells are presented in Fig. 7. For all three protocols (so called, “wound healing,” “anti-inflammatory,” and “analgesic” by the manufacturer), placing the Petri dish in the middle of the panel, 4 cm above the light source, resulted in a greater PpIX production compared with placing the sample directly above the LEDs Figs. 7(d) and 7(e). Furthermore, the “anti-inflammatory” protocol appeared to produce slightly higher PpIX levels, followed by the “wound healing” and “analgesic” protocols Figs. 7(d)–7(e), although differences between protocols were modest. PBM irradiation was applied prior to ALA administration, thereby excluding any contribution of PpIX-mediated photodynamic effects during light exposure.

The effects of these protocols on cell migration were also explored using a wound-healing (scratch) assay [Figs. 7(f)–7(i)]. Statistical analysis was performed using one-way ANOVA followed by Tukey’s multiple-comparisons test. Quantitative analysis demonstrated protocol-dependent differences in cell migration [Fig. 7(j)]. The “wound healing” protocol produced the strongest enhancement of wound closure and was significantly different from both the “anti-inflammatory” (P=0.00434) and “analgesic” (P=0.01661) protocols. By contrast, no statistically significant difference was observed between the “anti-inflammatory” and “analgesic” protocols (P=0.08988). No morphological signs of cytotoxicity were observed following PBM exposure. Phase-contrast microscopy demonstrated intact monolayers without increased detachment, rounding, or membrane disruption under any irradiation condition [Figs. 7(f)–7(i).

## 4 Discussion

The accurate and comprehensive characterization of the radiometric and spectral properties of light-emitting devices is a fundamental prerequisite for effective and reproducible phototherapy applications. This study addressed this critical need by developing and applying a robust methodology for the characterization of the irradiance generated at different wavelengths by a multipanel light-emitting device, the ATP38®. Our findings not only provide essential data for this specific device but, more importantly, establish a generalizable framework for similar precise characterization of diverse planar light sources used in biomedical and other fields.

The spectral characterization, as detailed in our results, provides one foundational feature of the light emitted by the device. Precisely determining the central wavelengths and their respective FWHM values is crucial, as the biological effects of light are highly wavelength-dependent.^2^^,^^13^ Variations in spectral output, even within nominally similar devices, temperature changes, or due to manufacturing deviations, can significantly impact therapeutic outcomes and device performance.^14^^,^^15^ The detailed approach, involving spectrophotometry and careful data processing, ensures that the spectral properties are accurately captured, providing reliable input for subsequent dose calculations and mechanistic studies.

The radiometric characterization, focusing on irradiance mapping, is central to determining the spatial distribution of light at the surface but, importantly, also within the illuminated tissue. A critical aspect of our methodology was the empirical correction for the power meter’s nonideal angular response. Photodetectors often exhibit a deviation from the ideal cosine response, especially at large illumination angles, which can lead to significant measurement inaccuracies if not addressed properly.^16^^,^^17^ These deviations frequently result from detector surface reflections (Fresnel), anti-reflection coatings, scattering, and microstructure, all reducing light detection at grazing angles. These effects effectively multiply the basic cos θ dependence by additional angular factors, so a detector’s angular response is often well modeled as $\cos^{m}(\theta )$. The fit of the measurements in Fig. 3(c) strongly supports this approach. Therefore, by characterizing and modeling this angular dependence with a $\cos^{m}(\theta )$ function, we ensured that the measured irradiance maps accurately reflect the true irradiance distribution, regardless of the incident angle. This meticulous correction is vital for obtaining reliable data, particularly when dealing with divergent light sources.

It should be noted that one limitation of the method proposed here is in relation to the relative values of the source dimensions as compared with the source–detector distance. Indeed, when the emitting surface dimensions are not negligible compared with the source–detector distance (i.e., no longer “point” sources), correcting the raw measurements as presented in this study requires a more sophisticated procedure than the relatively simple correction described above.

The subsequent fitting of the corrected 2D irradiance maps with a double 2D Gaussian surface model represents a powerful analytical step. This mathematical modeling transforms discrete measurement points into a continuous, high-resolution irradiance map. The high goodness of fit ($r^{2}>0.95$) achieved across all wavelengths underscores the accuracy and robustness of this fitting approach based on the hypothesis that the light is emitted according to a Gaussian profile. The ability to generate irradiance maps with arbitrary spatial resolution from these fits is invaluable for detailed dosimetry planning, allowing for precise spatial dose calculations that would be impractical with discrete measurements alone. Interestingly, and critically for the generalizability of this method, the fitted Gaussian surfaces enable the calculation of irradiance distributions at any working distance through a simple homothecy (scaling). This eliminates the need for repeated experimental measurements at every conceivable working distance, significantly streamlining the characterization process for devices intended for variable-distance applications.

The simulation of the three-panel planar configuration by superimposing individual panel contributions demonstrates the scalability and predictive power of our methodology. This capability is particularly important for clinical applications where devices are often used in multisource configurations, and understanding the combined light field is essential for optimizing treatment parameters.

The integration of our physical characterization with biological assays provides a crucial link between a device’s radiometric properties and its therapeutic efficacy. Importantly, the applied PBM protocols were not intended to induce cytotoxicity, and no evidence of irradiation-related cell damage was detected in the present experiments. Microscopic evaluation revealed preserved morphology and confluence following illumination. Moreover, PBM exposure preceded ALA incubation, preventing any photodynamic effects mediated by newly synthesized PpIX. Previous work using comparable irradiation parameters likewise demonstrated the absence of cell death, together with increased metabolic activity and cell number 24 h after treatment.^11^ These observations indicate that the biological responses observed here reflect PBM-driven modulation of cellular metabolism rather than irradiation-induced toxicity. Three light delivery protocols proposed by the manufacturer of the ATP38® have been considered. Our findings from the PpIX production and wound healing assays confirm that different PBM protocols, defined by their light modulation frequency, elicit distinct biological responses. The observation that the continuous-wave (“wound healing,” 0 Hz) protocol produced strong biological responses should be interpreted cautiously. Direct comparisons between pulsed and continuous-wave PBM remain limited, and available studies report heterogeneous and sometimes contradictory outcomes depending on irradiation parameters, biological models, and endpoints investigated.^18^^,^^19^ Consequently, it is currently difficult to attribute a general mechanistic advantage to one temporal delivery mode. The present findings therefore highlight the need for further systematic investigation of pulse structure and temporal dose delivery in PBM applications.

Furthermore, our results highlight the significant impact of sample placement on PBM outcomes. We found that placing the cell cultures in the middle of the panel had a more potent PBM effect on PpIX production compared to placing them directly above the LEDs. This finding must be considered in light of the irradiance maps generated in our physical characterization. The center of the panel, being a point of additive contributions from the multiple light sources, likely receives a more diffuse and uniform irradiance, or dose of light, across the sample area, whereas the area directly above the LEDs experiences higher, but potentially more heterogeneous irradiance distribution. The ability to generate accurate and uniform irradiance maps, as demonstrated in this study, is therefore indispensable for optimizing treatment protocols by ensuring a homogeneous and therapeutically effective dose delivered across the target area. These results may also reflect a bimodal dose-response pattern, often described as the Arndt–Schulz law.^20^^,^^21^ Specifically, when irradiance or exposure duration is below a certain threshold, no measurable biological response occurs. Conversely, excessive light intensity or prolonged illumination can also fail to elicit a beneficial PBM effect, resulting in a diminished or absent response.

The primary take-home message from this study is the development of a comprehensive and highly adaptable methodology for the spectral and radiometric characterization of light-emitting devices. This approach, encompassing precise spectral analysis, rigorous angular response correction, robust Gaussian fitting for continuous spatial mapping, and scalable simulation of multi-source configurations, is not limited to the ATP38® device. Instead, it provides a versatile blueprint that can be applied to most light-emitting devices where accurate light dose estimation is required, from other phototherapy systems to diagnostic tools or industrial light sources.

The irradiance maps generated by this methodology serve as crucial input parameters for light propagation simulations in biological tissues.^10^ Such simulations are indispensable for moving beyond surface irradiance measurements to truly understand the fluence rate, and consequently the absorbed dose, distribution within complex tissue structures, which is the ultimate determinant of biological response. Future work will leverage these characterized light fields to model the propagation of light in tissues, thereby enabling more precise and personalized phototherapy protocols.

## 5 Conclusion

This study showed a quantitative characterization of the irradiance produced by the ATP38® device across its different emitted wavelengths. Although our measurements of the irradiance have been performed at a working distance of 4 cm, the method presented in this article enables us to easily derive the illumination delivered at other distances. Concurrently, its spectral properties were precisely determined. The comprehensive methodology developed and applied herein, encompassing detailed spectral analysis, rigorous angular response correction for the detector, and advanced Gaussian fitting of irradiance maps, provides a robust and generalizable framework. This framework is highly adaptable for the radiometric and spectral characterization of most light-emitting devices where accurate light dose estimation is critical. The continuous irradiance maps derived from our fitting procedure are particularly valuable, offering the flexibility to calculate light distributions at arbitrary spatial resolutions and working distances through simple scaling. These precisely characterized irradiance maps, coupled with our biological efficacy findings, highlight the critical link between physical device properties and therapeutic outcome. These precisely characterized irradiance maps will serve as essential input parameters for subsequent studies focused on simulating light propagation within biological tissues, providing a foundation for more accurate and optimized phototherapy dosimetry.

## Data Availability

The data that support the findings of this study are available upon reasonable request.
